# Single Step In Situ Synthesis and Optical Properties of Polyaniline/ZnO Nanocomposites

**DOI:** 10.1155/2014/904513

**Published:** 2014-01-02

**Authors:** Deepali Sharma, B. S. Kaith, Jaspreet Rajput

**Affiliations:** ^1^Department of Chemistry, Sant Longowal Institute of Engineering and Technology, Longowal, Sangrur, Punjab 148106, India; ^2^Department of Chemistry, Dr. B. R. Ambedkar National Institute of Technology, Jalandhar, Punjab 144 011, India

## Abstract

Polyaniline/ZnO nanocomposites were prepared by in situ oxidative polymerization of aniline monomer in the presence of different weight percentages of ZnO nanostructures. The steric stabilizer added to prevent the agglomeration of nanostructures in the polymer matrix was found to affect the final properties of the nanocomposite. ZnO nanostructures of various morphologies and sizes were prepared in the absence and presence of sodium lauryl sulphate (SLS) surfactant under different reaction conditions like in the presence of microwave radiation (microwave oven), under pressure (autoclave), under vacuum (vacuum oven), and at room temperature (ambient condition). The conductivity of these synthesized nanocomposites was evaluated using two-probe method and the effect of concentration of ZnO nanostructures on conductivity was observed. X-ray diffraction (XRD), scanning electron microscopy (SEM), transmission electron microscopy (TEM), Fourier transform infrared spectroscopy (FTIR), and UV-visible (UV-VIS) spectroscopy techniques were used to characterize nanocomposites. The optical energy band gap of the nanocomposites was calculated from absorption spectra and ranged between 1.5 and 3.21 eV. The reported values depicted the blue shift in nanocomposites as compared to the band gap energies of synthesized ZnO nanostructures. The present work focuses on the one-step synthesis and potential use of PANI/ZnO nanocomposite in molecular electronics as well as in optical devices.

## 1. Introduction

With the advent of advancement in the field of nanoscience and nanotechnology in the recent years, nanocomposites of different metals and conducting polymers or metal oxide-polymer have become an important class of materials. These materials find potential applications as sensors, UV detectors, catalysts, biosensors, and piezoelectronic materials [1–4]. As with conventional composites, the properties of nanocomposites can display synergistic improvements over those of the component phases individually. However, by reducing the physical dimension(s) of the phase(s) down to the nanometer length scale, unusual and often enhanced properties can be realized. An important microstructural feature of nanocomposites is their large ratio of interphase surface area to volume. The nanocomposites differ from pure polymers and inorganic metal oxide nanoparticles in some physical and chemical properties.

Among various metal oxide nanoparticles, ZnO is a key technological and multifunctional inorganic material with unaccountable applications such as sensors, optical, electronic, magnetic, catalytic and detection of biological molecules [5–11]. It is a versatile functional material that has a diverse group of growth morphologies, such as nanocombs, nanorings, nanohelixes/nanosprings, nanobelts, nanobeads, nanowires, and nanocages [12–17]. These unique nanostructures unambiguously demonstrate that ZnO has the richest family of nanostructures among all materials, both in structures and in properties. It has a direct band gap of 3.37 eV and high exciton binding energy of 60 meV at room temperature [18–20].

Among different conducting polymers, polyaniline (PANI) is one of the most widely studied conducting polymers over the past 50 years and thus has received great attention due to its ease of synthesis, environmental stability, electrical, optical and electrochemical properties, and simple doping/dedoping chemistry [21]. It was first synthesized in 1862 and has been studied since 1980s [22]. Polyaniline can exist in various oxidation states. The three major oxidation states are leucoemeraldine (white/clear and colourless), emeraldine (green for emeraldine salt and blue for emeraldine base), and pernigraniline (blue/violet). Among the above three oxidation states, the fully reduced state is leucoemeraldine with *n* = 1, *m* = 0. Pernigraniline is the fully oxidized state with *n* = 0, *m* = 1. The emeraldine (*n* = *m* = 0.5) form of polyaniline is also referred to as emeraldine base (EB) and is neutral. On doping, it changes to emeraldine salt (ES) with the imine nitrogens protonated by an acid. Emeraldine base (EB) is considered to be the most useful form of aniline due to its high stability at room temperature. On doping emeraldine base (EB) with nonoxidising protonic acids such as HCl, H_2_SO_4_, or organic acids (p-toluene sulfonic acid), it is converted into emeraldine salt (ES) form which is electrically conducting [23, 24]. Polyaniline can be easily synthesized either chemically or electrochemically from acidic aqueous solutions [25]. The most common synthesis of polyaniline is by oxidative polymerization with ammonium peroxodisulfate as an oxidant [26].

Various methods have been reported for the synthesis of nanocomposites. The most common method involves the oxidative polymerization of aniline. In this, aqueous aniline is dissolved in 1 M HCl regulating the temperature at 0°C followed by the addition of oxidant (ammonium peroxydisulfate). The use of surfactants assures a good dispersion of metal oxide nanoparticles in the polymer along with embedding them in the growing polymer during polymerization [27, 28]. Chemical methods based on the in situ sol gel polymerization method allow single-step synthesis of polymer inorganic nanocomposites in the presence of polymer or monomer [29]. It is possible to manipulate the organic or inorganic interfacial interactions at various molecular and nanometer length scales using this method, resulting in homogeneous polymer inorganic nanocomposites structures and thus, overcoming the problem of nanoparticle agglomeration [30–32].

In the present work, polyaniline (PANI)/ZnO nanocomposites have been synthesized by a single-step process by loading different weights of ZnO nanostructures synthesized in the presence and absence of sodium lauryl surfactant (SLS) and characterized for their structural and optical properties. Further, the conductivity of the nanocomposites has been evaluated using two-probe method.

## 2. Experimental

### 2.1. Materials and Methods

#### 2.1.1. Chemicals

ZnSO_4_·7H_2_O (SdFine), NaOH (SdFine), aniline (SdFine), HCl (35% GR), ammonium peroxidisulfate (MERCK), sodium lauryl sulphate (SdFine), methanol (MERCK, 99%), and as-synthesized ZnO nanoparticles were used. Double-distilled water was employed.

#### 2.1.2. Equipments

Microwave (LG Model no. MB394AA), vacuum oven (Navyug India Q-5247), autoclave (LABCO-India ISO-9001), UV-visible spectrophotometer (Systronics Double Beam UV-Vis Spectrophotometer: 2201), vacuum filter (HARCO), and ELIX 3Millipore were used.

### 2.2. Synthesis of ZnO Nanostructures

#### 2.2.1. Surfactant Free Microwave Synthesis

Initially, 50 mL of zinc sulphate heptahydrate (ZnSO_4_·7H_2_O) and 50 mL of sodium hydroxide (NaOH) solutions in 1 : 4 molar ratio were prepared and were mixed together in a reaction flask, followed by vigorous stirring for 15 min at ambient temperature. The reaction was further carried out under the influence of microwave radiations by placing the reaction flask in the microwave for 2 min. The reaction flask was cooled to room temperature and the product was filtered and washed with deionized water followed by drying at 40°C.

#### 2.2.2. Surfactant Assisted Synthesis under Different Reaction Conditions

In a typical procedure, 100 mL of zinc sulphate heptahydrate (ZnSO_4_·7H_2_O) solution and 100 mL of sodium lauryl sulphate (surfactant) solution were mixed with 100 mL of sodium hydroxide (NaOH) solution in 1 : 0.4 : 4 molar ratio and stirred vigorously for 15 min. This was followed by exposure to microwave irradiation for 2 min. The white product obtained was washed 5-6 times with distilled water and ethanol and dried at 40°C. Similar procedure was followed to obtain the product under other reaction conditions like under pressure (UP, pressure: 5 psi), under vacuum (UV, vacuum: 160 mmHg), and at room temperature (RT).

### 2.3. Synthesis of Polyaniline (PANI)

0.25 M aniline was added to the mixture of 0.175 M sodium lauryl sulphate (SLS) and 0.75 M HCl at 0°C. The temperature of the ice bath was maintained at 0 ± 1°C. The resulting mixture was stirred for 1 h maintaining the above temperature. It was followed by the addition of 1.2 g of ammonium persulfate (APS) dissolved in 20 mL distilled water. The resulting mixture was stirred vigorously for 1 h. Polyaniline was precipitated with methanol. The green coloured product was obtained by vacuum filtration and washed several times with double distilled water and dried at 45°C for 8 h. The dried and fine powdered green coloured product was pressed in the form of pellet using hydraulic press.

### 2.4. Preparation of Polyaniline (PANI)/ZnO Nanocomposites

To prepare nanocomposite, 0.25 M aniline was added to 0.175 M sodium lauryl sulphate (SLS) and 0.75 M HCl at 0°C. The temperature of the ice bath was maintained at 0 ± 1°C. The resulting reaction mixture was stirred for 1 h maintaining the above temperature. This was followed by the addition of different percentages of ZnO nanostructures synthesized in the absence and presence of sodium lauryl sulphate (SLS) surfactant under different reaction conditions. 1.2 g of ammonium persulfate (APS) dissolved in 20 mL distilled water was added slowly to the reaction mixture with constant stirring. The reaction mixture turned viscous and green and was stirred for 1 h. The nanocomposite formed was precipitated using methanol. Finally, the green coloured product was obtained by vacuum filtration and washed several times with double distilled water and dried at 45°C for 8 h. The dried and fine powdered green coloured product was pressed in the form of pellets using hydraulic press and characterized using XRD, SEM, TEM, FTIR, and UV-Vis spectrophotometric techniques.

### 2.5. Conductivity Measurement of Polyaniline (PANI)/ZnO Nanocomposite

The conductivity measurement of the dried pellets was carried out using two-probe method. All the measurements were done at room temperature (30°C) at an applied voltage of 20 volts. Measurements were taken after 15 min of applying voltage.

## 3. Results and Discussion

The polymerization of aniline was carried out in the aqueous solution in the presence of sodium lauryl sulphate and hydrochloric acid at low temperature (0 ± 1°C). Intial stages of polymerization resulted in the formation of aniline dodecylsulphonic acid and aniline hydrochloric acid which enhanced the solubility of polymer and provided a conducting polymer structure. As soon as ammonium persulfate (APS) was added, the colour of the reaction mixture changed from white to green and finally to dark green colouration. The different weight percentages of ZnO nanostructures synthesized under different reaction conditions were added prior to adding APS. Thus, PANI/ZnO nanocomposite was formed during the polymerization process.

**Scheme 1:**
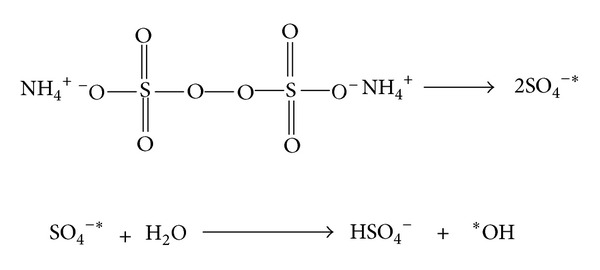


Polymerization of the aniline takes place through (Scheme 1) proposed mechanism.

The *OH generated from APS resulted in the formation of aniline cation radical which initiated the polymerization process.

**Scheme 2:**
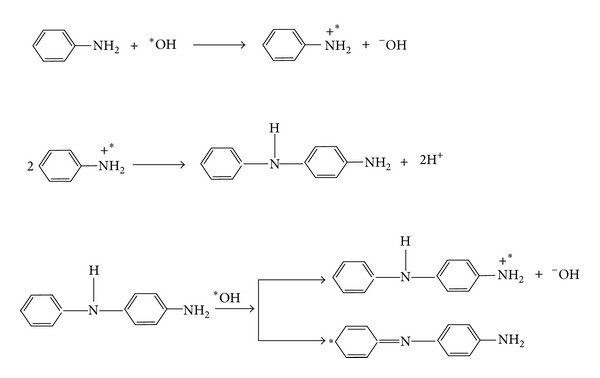


*Initiation*. See Scheme 2.

**Scheme 3:**
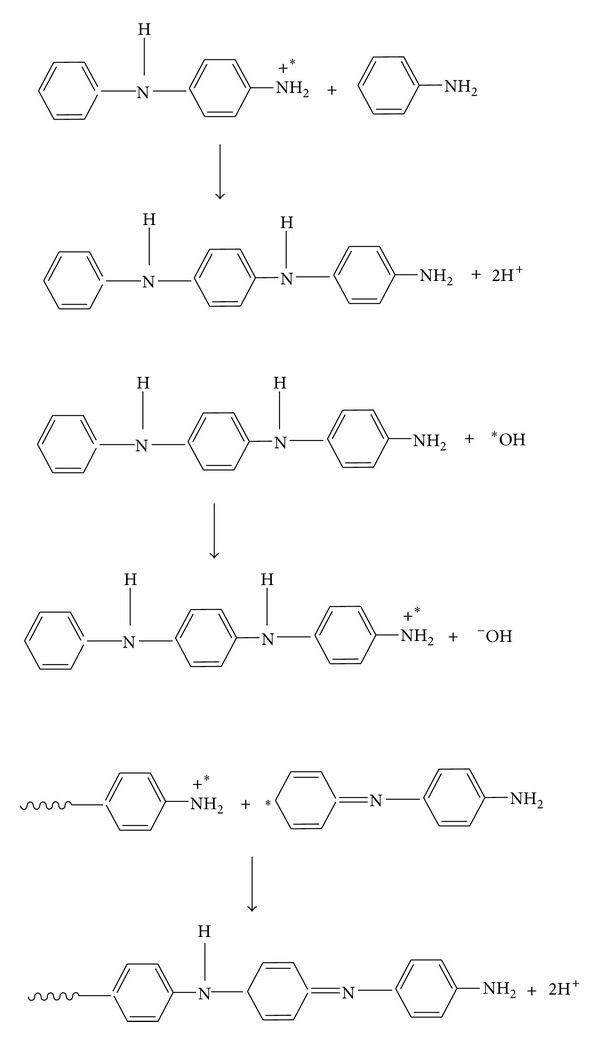


*Propagation*. See Scheme 3.

### 3.1. Characterization

Characterization was carried out with X-ray diffractometer (PANalytical), Scanning electron microscope (JSM-6610LV), transmission electron microscope (Technai G^2^ X 9900), Fourier-transform infrared spectrometer (FTIR, Model 1600 Perkin-Elmer), and UV-visible spectrophotometer (SYSTRONICS DOUBLE BEAM UV-VIS Spectrophotometer: 2201).

#### 3.1.1. X-Ray Diffraction (XRD) Studies

**Figure 1:**
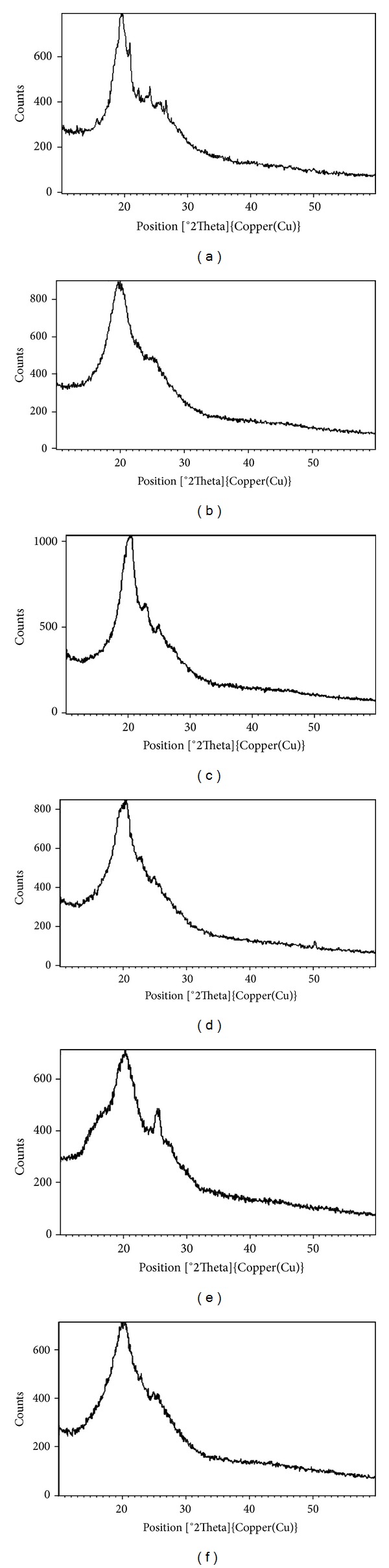
XRD Spectra of (a) polyaniline (PANI), (b) PANI/60% ZnO-SF-MW, (c) PANI/60% ZnO-SLS-MW, (d) PANI/40% ZnO-SLS-UP, (e) PANI/60% ZnO-SLS-UV, and (f) PANI/40% ZnO-SLS-RT nanocomposites.

Figure 1(a) represents the XRD pattern of polyaniline. A maximum peak at 2*θ* = 20.9° can be assigned to the scattering from the polyaniline chains at interplanar spacing [33]. From the XRD pattern it was observed that the synthesized polyaniline was partially crystalline, that is, amorphous in nature. Figures 1(b), 1(c), 1(d), 1(e), and 1(f) show the XRD patterns of PANI/ZnO nanocomposites. In Figure 1(b), 60% ZnO nanoparticles of spherical morphology synthesized in the absence of surfactant have been incorporated into the polymer (PANI) matrix.  Figure 1(c) shows the incorporation of 60% ZnO nanoparticles synthesized using sodium lauryl sulphate (SLS) surfactant under microwave condition. In Figure 1(e), a sharp peak around 2*θ* = 25.6° appeared in the nanocomposite. A normal ZnO peak appears around 2*θ* = 30.0°. A shift in the peak was observed to lower angle. It was inferred from the pattern that ZnO nanostructures interacted with the chains of polyaniline. Figures 1(d) and 1(f) show the physical interaction of the 40% ZnO nanostructures synthesized using SLS under pressure and at room temperature, with the polymer chains.

The coherence length (CL) of PANI and PANI/ZnO nanocomposites was measured using Scherrer's equation:

(1) $$\begin{array}{c} \text{CL}=\frac{\lambda k}{\beta \;\cos\;\theta }, \end{array}$$

where *λ* is wavelength (1.54 Å); *k* is the constant (0.9); *β* = full width at half maxima (FWHM); *θ* is the wide angle XRD peak position.

The data obtained after applying Scherrer's equation has been given in Table 1.

It has been observed that the coherence length (CL) of PANI/ZnO nanocomposites was higher in comparison to that of PANI (Table 1). Thus, higher coherence length indicated higher crystallinity and crystalline coherence which further contributed to higher conductivity of nanocomposites as compared to PANI [34, 35].

**Figure 2:**
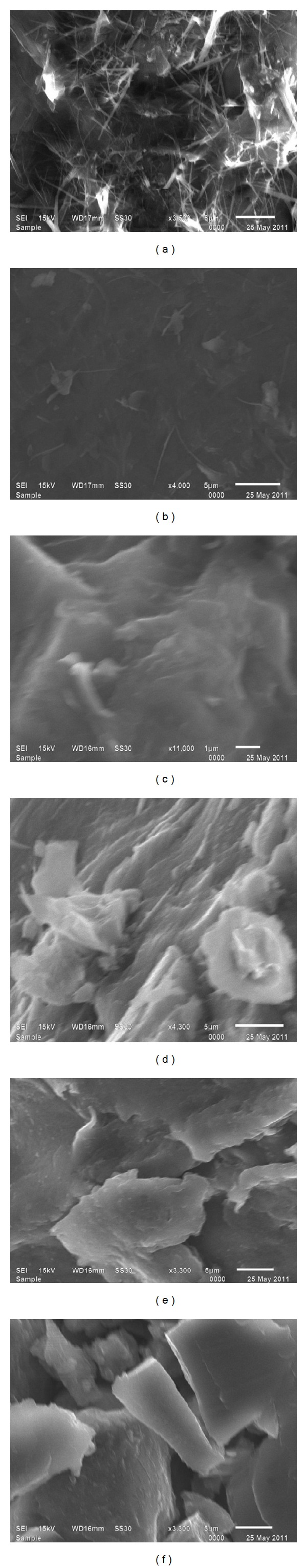
SEM micrographs of (a) polyaniline (PANI), (b) PANI/60% ZnO-SF-MW, (c) PANI/60% ZnO-SLS-MW, (d) PANI/40% ZnO-SLS-UP, (e) PANI/60% ZnO-SLS-UV, and (f) PANI/40% ZnO-SLS-RT nanocomposites.

In the case of nanocomposites, the calculated coherence length depends on how the ZnO nanoparticles are embedded in the polymer matrix and are linked to the polymeric chains. In the present case, ZnO-SLS-MW was reported to have high coherence length value as the nanorods linked well with the polymeric chains (Figure 2(c)). It has been observed from the SEM image (Figure 2(b)) that the spherical shaped particles dispersed well within the polymer matrix. Due to formation of nanoneedles of length 120 nm in the case of ZnO-SLS-RT, they lead to good coherence value. The nanoplates formed in the case of ZnO-SLS-UV linked with the polymer chains but not in ordered manner. Similarly, nanoflowers formed via ZnO-SLS-UP seemed to overlap while linking with the polymer chains (Figure 2(d)). Thus, it could be concluded that coherence length is much dependent on how the nanoparticles are arranged in the polymer matrix rather than being dependent on morphology, size, and surface area.

#### 3.1.2. Scanning Electron Microscopy (SEM) Studies

Figure 2(a) shows the surface morphology of the as-synthesized polyaniline. Figures 2(b)–2(f) are SEM images of the nanocomposite with varying percentage of ZnO nanostructures. It is evident from the SEM micrographs that the morphology of polyaniline has changed with the introduction of ZnO nanostructures of different morphologies. Figures 2(b) and 2(c) depict the uniform distribution of spherical and nanorod shaped ZnO into the polymer matrix, respectively. Figure 2(d) shows the incorporation of ZnO nanoflowers synthesized using SLS under pressure into the polymer matrix. Thus, it was interpreted that there was an effective interaction of ZnO nanostructures of varied morphology with polyaniline matrix.

#### 3.1.3. Transmission Electron Microscopy (TEM) Studies

**Figure 3:**
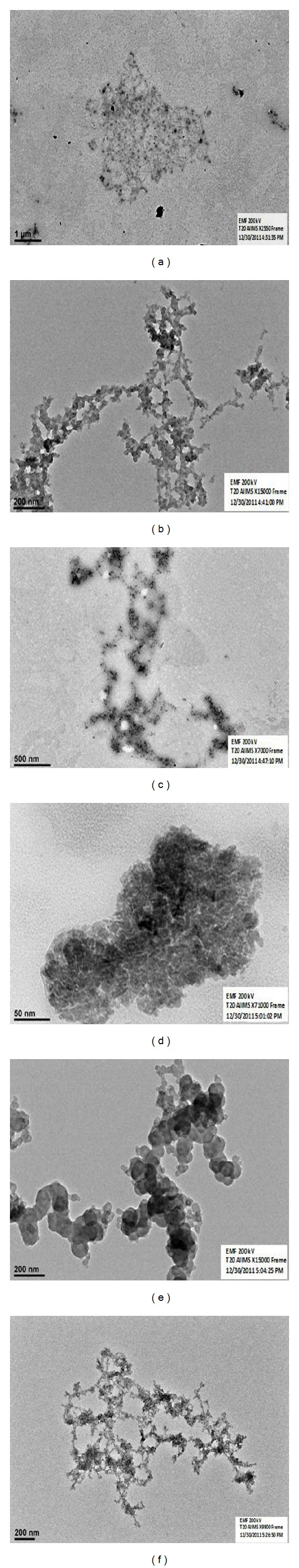
TEM images of (a) polyaniline (PANI), (b) PANI/60% ZnO-SF-MW, (c) PANI/60% ZnO-SLS-MW, (d) PANI/40% ZnO-SLS-UP, (e) PANI/60% ZnO-SLS-UV, and (f) PANI/40% ZnO-SLS-RT nanocomposites.

Figure 3(a) represents the TEM image of polyaniline network containing chains of the polymer whereas Figures 3(b)–3(e) represent the TEM images of PANI/ZnO nanocomposites containing different weight percentages of ZnO nanostructures synthesized via surfactant free and surfactant assisted methods.  Figure 3(b) is a TEM image of nanocomposite containing 60% ZnO nanostructures synthesized using microwave method in the absence of surfactant, SLS. It has been observed that spherical ZnO nanoparticles in the size range of 20–25 nm have been dispersed in the polymer matrix. The dark spots in the TEM image are the nanoparticles. Figures 3(c) and 3(d) show the TEM images where ZnO nanostructures synthesized in the presence of SLS under microwave (60% ZnO) and under pressure (40% ZnO) have been well entrapped in the chains of polyaniline. Similarly, in the Figures 3(e) and 3(f), 60% of ZnO nanostructures synthesized under vacuum (UV) and 40% of ZnO nanostructures synthesized at room temperature (RT) methods have been embedded in the matrix of polyaniline. Thus, Figures 3(b)–3(e) indicate that the surface of ZnO nanostructure has interaction with the PANI chains.

#### 3.1.4. Fourier-Transform Infrared Spectroscopy (FTIR) Studies

**Figure 4:**
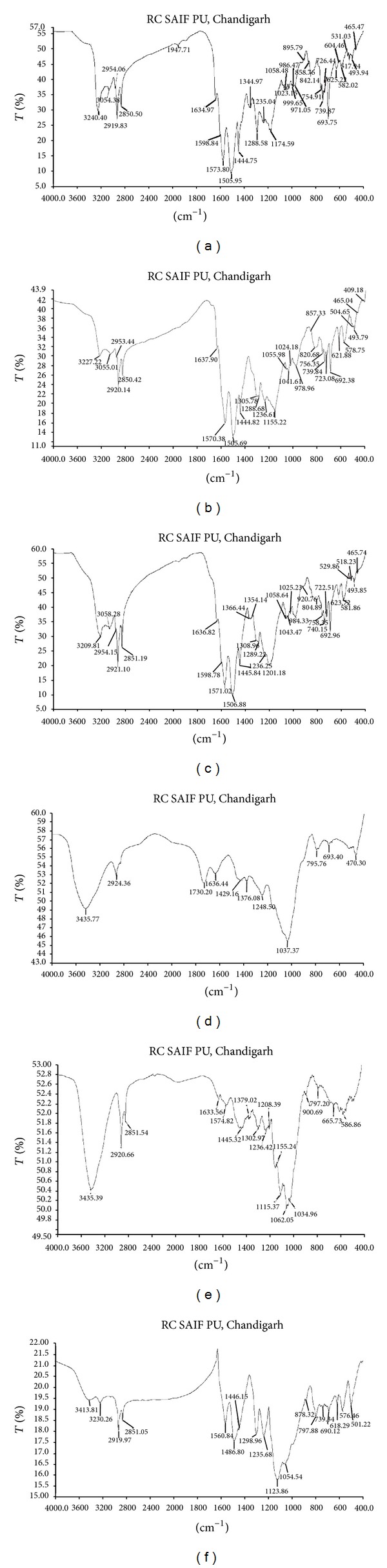
FTIR spectra of (a) polyaniline (PANI), (b) PANI/60% ZnO-SF-MW, (c) PANI/60% ZnO-SLS-MW, (d) PANI/40% ZnO-SLS-UP, (e) PANI/60% ZnO-SLS-UV, and (f) PANI/40% ZnO-SLS-RT nanocomposites.

Figure 4(a) illustrates the FTIR spectrum of polyaniline and Figures 4(b)–4(f) represent the FTIR spectra of nanocomposites, respectively. In Figure 4(a), the peaks at 1573.8 cm^−1^ and 1444.75 cm^−1^ correspond to C=C stretching of quinoid and benzenoid rings, respectively. A sharp peak at 1288.58 cm^−1^ is characteristic of C–N stretching whereas a peak at 3240.40 cm^−1^ is of N–H stretching mode. A peak at 3054.38 cm^−1^ belongs to C–H stretching. –CH_2_ stretching occurs as a sharp peak at 2919.83 cm^−1^. The peaks at 517 cm^−1^ and 693.75 cm^−1^ correspond to C–Cl stretching and NH_2_ wagging, respectively. In Figure 4(b), there is a shift in the frequency of C=C stretching of quinoid ring from 1573.8 cm^−1^ to 1570.38 cm^−1^. N–H stretching mode has moved to lower frequency (3227.22 cm^−1^) thereby decreasing the intensity of the peak. The peak present in Figure 4(a) at 517 cm^−1^ has vanished in Figure 4(b). This shows that there is bond formation between ZnO and amine group of polyaniline. Similarly, in Figure 4(c), C=C stretching of quinoid ring occurs at 1571.02 cm^−1^ and N–H stretching mode at 3209.81 cm^−1^. This shift in the frequencies confirms the formation of bond between ZnO and PANI and finally nanocomposite. In Figures 4(d) and 4(e), a broad peak occurs at 3435.77 cm^−1^ and 3435.39 cm^−1^, respectively. This belongs to N–H stretching mode. A weak peak of –CH_2_ stretching occurs at 2924.36 cm^−1^. This occurs as a sharp peak at 2920.66 cm^−1^ in Figure 4(e). The other peaks occurring in Figure 4(a) at 3054.38 cm^−1^, 1573.8 cm^−1^, and 517 cm^−1^ have vanished in the spectrum of Figure 4(d). NH_2_ wagging occurs as a very weak peak at 693.40 cm^−1^. In Figure 4(f), there is a shift in the N–H stretching mode to lower frequency (very weak band at 3413.81 cm^−1^). C=C stretching of quinoid has moved to 1560.84 cm^−1^ whereas, for benzenoid ring, the stretching frequency is at 1486.80 cm^−1^ as compared to that in Figure 4(a). Thus, the above spectra (Figures 4(b)–4(f)) confirm the formation of PANI/ZnO nanocomposites [33].

#### 3.1.5. UV-Visible (UV-VIS) Studies

**Figure 5:**
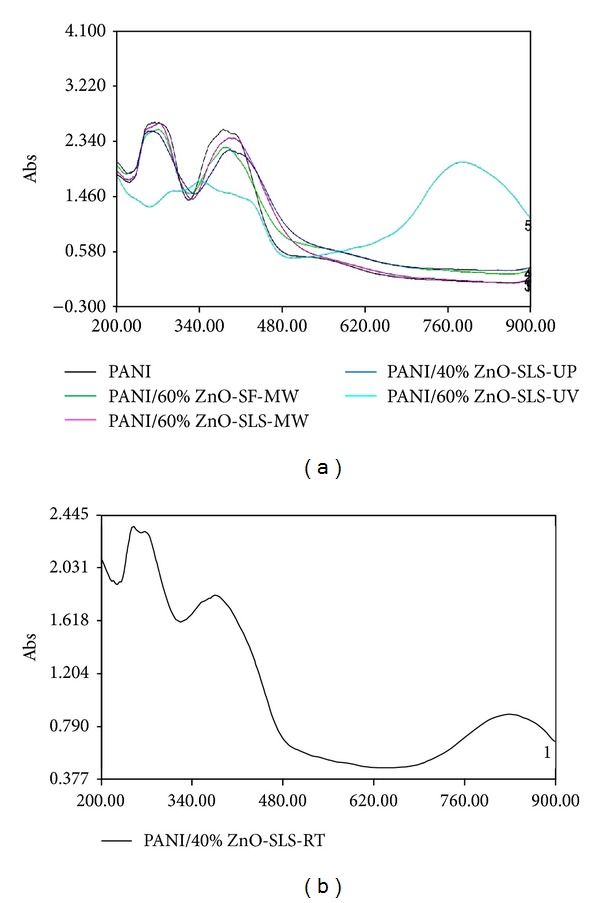
(a) UV-visible spectra of PANI and PANI/ZnO nanocomposites in chloroform. (b) UV-visible spectrum of PANI/ZnO nanocomposite in chloroform.

Figures 5(a) and 5(b) represent the UV-VIS absorption spectra of the synthesized polyaniline (PANI) and polyaniline (PANI)/ZnO nanocomposites. In Figure 5(a), polyaniline (PANI) exhibits two broad absorption peaks at 253.2 nm and 379.2 nm. This peak corresponds to the *π*-π* transition of the benzenoid ring and constitutes the typical emeraldine salt spectrum. A little red shift was observed for the nanocomposites containing 60% ZnO nanostructures (synthesized in the absence and presence of surfactant SLS under microwave) and 40% ZnO nanostructures (synthesized using SLS under pressure), respectively. This red shift was due to the interaction of polyaniline with ZnO. In the absorption spectrum of nanocomposite containing 60% ZnO nanostructures (synthesized using SLS under vacuum), a large red shift was observed and the broad peaks appeared at 298.0 nm, 342.7 nm, and 776.8 nm. The peak at 776.8 nm could be assigned to the polaron band transitions. The appearance of this peak in the absorption spectra showed that the polymer chains which have coiled conformation (less conjugation) in chloroform extended causing dispersion and strong interaction between adjacent polarons. Also, it was confirmed that in this case there was strong interaction of ZnO nanoparticles with the polyaniline. Similarly, in Figure 5(b), a large red shift was observed and a broad peak appeared at 821.0 nm in addition to two other peaks.

The band gap energy (*E*_*g*_) of the nanocomposites was determined by substituting the value of the absorption peak at a given wavelength in the following equation [36]:

(2) $$\begin{array}{c} E_{g}=hv_{g}=\frac{hc}{\lambda_{g}}, \end{array}$$

where *h* = 4.14 × 10^−15^ eVs; *c* = 2.99 × 10^8^ m/s; *λ*_*g*_ is the wavelength at maximum absorption of each nanocomposite. The *E*_*g*_ values have been reported in the Table 2. The blue shift was observed in the case of PANI/ZnO nanocomposites as compared to ZnO nanostructures. As the content of ZnO nanostructures was increased in the polymer matrix, there was a red shift. This was noticed in PANI/60% ZnO-SF-MW, PANI/60% ZnO-SLS-MW, and PANI/40% ZnO-SLS-UP. Similar result was observed in PANI/60% ZnO-SLS-UV and PANI/40% ZnO-SLS-RT. Thus, the optical band gap energy is found to be dependent on the composition of nanocomposites [37].

**Figure 6:**
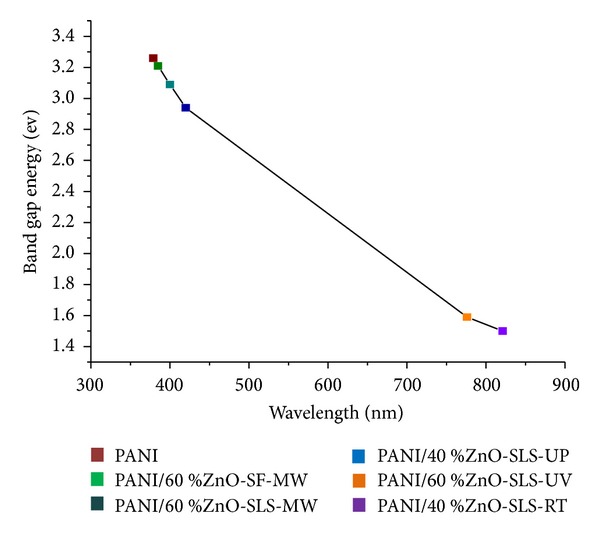
Variation of optical band gap energy (*E*_*g*_) with wavelength at maximum absorption (*λ*_*g*_) of nanocomposites.

Further, it was observed that, with increase in the wavelength at maximum absorption, the band gap energy (*E*_*g*_) values decreased (Figure 6).

### 3.2. Conductivity Measurement

The conductivities of as-synthesized polyaniline and PANI/ZnO nanocomposites with different amounts of ZnO nanostructures were measured by two-probe method. The results indicated that, as the content of the ZnO nanostructures was increased, the conductivity of the nanocomposite increased as compared with that of polyaniline. 20% to 80% of the initial weight of ZnO nanostructures synthesized in absence and presence of surfactant (sodium lauryl sulphate, SLS) under different reaction conditions were taken. All the measurements were done at room temperature (30°C) at an applied voltage of 20 volts. Measurements were taken after 15 min of applying voltage.

In two-probe instrument, electrodes were of Al foil of thickness 1 mm and area 1 cm^2^.

Conductivity (*σ*_dc_) was calculated using the following formula:

(3) $$\begin{array}{c} \sigma_{\text{dc}}=\frac{l}{Ra}, \end{array}$$

where *l* is the sample thickness (thickness of the pellet); *R* is the resistance; *a* is the cross-sectional area of electrode.

Conductivity data of polyaniline/ZnO nanocomposites has been presented in Table 3.

The results indicated that, as the content of the ZnO nanostructures was increased, the conductivity of the nanocomposite increased as compared with that of polyaniline. The addition of ZnO nanostructures in the polmer matric enhanced the conductivity of the polymer.

The conductivity was found to increase with the embedment of PANI with ZnO nanostructures. Initially, with increase in weight % of ZnO nanostructures, the conductivity of nanocomposites was found to increase. However, after reaching the maximum conductivity with optimum ZnO nanostructure concentration, further increase in ZnO nanoparticles resulted in decreased conductivity. This can be explained on the basis that high concentration of ZnO nanoparticles hinders the carrier transport between the different conjugated chains of polyaniline (PANI) [38]. The existence of interaction between PANI and ZnO nanostructures leads to the reduction of the conjugated lengths in the PANI chains. It has been observed that, in most of the cases, embedment of 60% ZnO nanostructures in the PANI matrix gave optimum conductivity values. The order of the conductivity found was

PANI/ZnO-SLS-MW > PANI/ZnO-SLS-UV > PANI/ZnO-SF-MW > ZnO-SLS-UP > PANI/ZnO-SLS-RT.

## 4. Conclusion

PANI/ZnO nanocomposites were synthesized via in situ oxidative polymerization of aniline monomer. Different weights of ZnO nanostructures prepared in the absence and presence of surfactant were added to the aniline prior to polymerization. The surface morphology changed with the addition of ZnO nanostructures. This is well evident from the SEM images of the nanocomposites. The surfactant sodium lauryl sulphate (SLS) was added to the aniline solution. This acted as a stabilizer and contained amine group which was grafted on the growing polymer (PANI) chains. Moreover, it assured a good dispersion of ZnO nanoparticles in the PANI matrix along with embedding them in the polymer chains. The surfactant also promotes the micelle formation and oxidation reaction. This is well represented in the FTIR spectra of polyaniline and nanocomposites. The UV-visible spectra demonstrated the shifting and change in the intensity of the peaks which confirmed the effective interaction of ZnO nanostructures with the polyaniline through the hydrogen bonding between the imine group (–NH) of PANI and hydroxyl (–OH) group of ZnO nanostructures. The calculated optical band gap energy values of nanocomposites were found to be dependent on the weight percent of ZnO nanostructures embedded in the polymer matrix. The observations show that PANI/ZnO nanocomposites can be used potentially in molecular electronics and optical devises. It was concluded that the conductivity of ZnO nanocomposites initially increased and then decreased with the increase in the content of ZnO nanostructures due to the fact that increased % of ZnO nanostructures hinders the carrier transport between the different conjugated chains of polyaniline (PANI).

## Figures and Tables

**Table 1:** Measurement of coherence length of PANI/ZnO nanocomposites.

Sample	Position [°2 Th]	FWHM [°2 Th]	d-spacing (Å)	Coherence length (nm)	*σ*_dc_, *S* cm^−1^
PANI	19.6234	0.9368	4.52399	16.9	4.5 × 10^−14^
PANI/60% ZnO-SF-MW	20.4360	0.7220	4.23657	21.7	1.82 × 10^−13^
PANI/60% ZnO-SLS-MW	23.0113	0.6691	3.86503	23.6	4.2 × 10^−13^
PANI/40% ZnO-SLS-UP	20.4430	0.9116	4.34083	17.3	1.15 × 10^−13^
PANI/60% ZnO-SLS-UV	25.6006	0.9183	3.47681	17.5	2.9 × 10^−13^
PANI/40% ZnO-SLS-RT	20.6597	0.8160	4.29579	19.2	2.07 × 10^−13^

**Table 2:** Band gap energy of ZnO nanoparticles and PANI/ZnO nanocomposites.

Sample	Morphology of ZnO nps	Size (avg. Dia nm) or length (nm) *d*_TEM_, ZnO nps	Band gap energy of ZnO nps (eV)	Band gap energy of PANI/ZnO nanocomposite (eV)
PANI	—	—	—	3.26
PANI/60% ZnO-SF-MW	Spherical	10–15 nm	3.61	3.21
PANI/60% ZnO-SLS-MW	Nanorods	*L* = 90 nm	3.48	3.09
PANI/40% ZnO-SLS-UP	Nanoflowers	Dia = 12–15 nm, *L* = 120 nm	3.61	2.94
PANI/60% ZnO-SLS-UV	Nanoplates	Dia = 20 nm, *L* = 150 nm	3.48	1.59
PANI/40% ZnO-SLS-RT	Nanoneedles	Dia = 10–12 nm, *L* = 120 nm	3.48	1.50

**Table 3:** Conductivity data of polyaniline/ZnO nanocomposites.

S. no.	Sample	% ZnO NPS	Current (*I*), amperes	Resistance (R), OHM	Sample thickness (*l*), cm	*σ*_dc_, *S*/cm
1	PANI	—	8 ∗ 10^−12^	2.5 ∗ 10^12^	0.088	4.5 ∗ 10^−14^
2	PANI/ZnO (in absence of surfactant)	20	9 ∗ 10^−12^	2.22 ∗ 10^12^	0.093	5.3 ∗ 10^−14^
3	PANI/ZnO (in absence of surfactant)	40	18 ∗ 10^−12^	1.11 ∗ 10^12^	0.147	1.6 ∗ 10^−13^
4	PANI/ZnO (in absence of surfactant)	60	15 ∗ 10^−12^	1.33 ∗ 10^12^	0.191	1.82 ∗ 10^−13^
5	PANI/ZnO (in absence of surfactant)	80	7 ∗ 10^−12^	2.85 ∗ 10^12^	0.004	1.79 ∗ 10^−15^
6	PANI/ZnO (in presence of surfactant, SLS, under microwave)	20	25 ∗ 10^−12^	0.80 ∗ 10^12^	0.195	3.1 ∗ 10^−13^
7	PANI/ZnO (in presence of surfactant, SLS, under microwave)	40	21 ∗ 10^−12^	0.95 ∗ 10^12^	0.109	1.46 ∗ 10^−13^
8	PANI/ZnO (in presence of surfactant, SLS, under microwave)	60	35.2 ∗ 10^−12^	0.57 ∗ 10^12^	0.19	4.2 ∗ 10^−13^
9	PANI/ZnO (in presence of surfactant, SLS, under microwave)	80	5 ∗ 10^−12^	4.0 ∗ 10^12^	0.143	4.55 ∗ 10^−14^
10	PANI/ZnO (in presence of surfactant, SLS, under pressure)	20	11 ∗ 10^−12^	1.82 ∗ 10^12^	0.129	9.1 ∗ 10^−14^
11	PANI/ZnO (in presence of surfactant, SLS, under pressure)	40	10 ∗ 10^−12^	2.0 ∗ 10^12^	0.181	1.15 ∗ 10^−13^
12	PANI/ZnO (in presence of surfactant, SLS, under pressure)	60	6 ∗ 10^−12^	3.33 ∗ 10^12^	0.180	6.9 ∗ 10^−14^
13	PANI/ZnO (in presence of surfactant, SLS, under pressure)	80	4 ∗ 10^−12^	5.0 ∗ 10^12^	0.150	3.8 ∗ 10^−14^
14	PANI/ZnO (in presence of surfactant, SLS, under vacuum)	20	16 ∗ 10^−12^	1.25 ∗ 10^12^	0.115	1.17 ∗ 10^−13^
15	PANI/ZnO (in presence of surfactant, SLS, under vacuum)	40	11 ∗ 10^−12^	1.81 ∗ 10^12^	0.199	1.40 ∗ 10^−13^
16	PANI/ZnO (in presence of surfactant, SLS, under vacuum)	60	24 ∗ 10^−12^	0.833 ∗ 10^12^	0.194	2.9 ∗ 10^−13^
17	PANI/ZnO (in presence of surfactant, SLS, under vacuum)	80	18 ∗ 10^−12^	1.11 ∗ 10^12^	0.114	1.3 ∗ 10^−13^
18	PANI/ZnO (in presence of surfactant, SLS, at room temperature)	20	9 ∗ 10^−12^	2.22 ∗ 10^12^	0.150	8.6 ∗ 10^−14^
19	PANI/ZnO (in presence of surfactant, SLS, at room temperature)	40	13 ∗ 10^−12^	1.53 ∗ 10^12^	0.249	2.07 ∗ 10^−13^
20	PANI/ZnO (in presence of surfactant, SLS, at room temperature)	60	9 ∗ 10^−12^	2.22 ∗ 10^12^	0.10	5.7 ∗ 10^−14^
21	PANI/ZnO (in presence of surfactant, SLS, at room temperature)	80	8 ∗ 10^−12^	2.5 ∗ 10^12^	0.11	5.6 ∗ 10^−14^
